# Mannose-binding lectin does not explain the dismal prognosis after an acute coronary event in dysglycaemic patients. A report from the GAMI cohort

**DOI:** 10.1186/s12933-022-01562-0

**Published:** 2022-07-08

**Authors:** Sara Meziani, Giulia Ferrannini, Mette Bjerre, Troels K. Hansen, Viveca Ritsinger, Anna Norhammar, Viveca Gyberg, Per Näsman, Lars Rydén, Linda G. Mellbin

**Affiliations:** 1 Department of Medicine Solna, Karolinska Institutet, Solnavägen 1, 171 76 Stockholm, Sweden; 2 Medical/Steno Aarhus Research Laboratory, Department of Clinical Medicine, Aarhus University, Aarhus, Denmark; 3 Steno Diabetes Center Aarhus, Aarhus University Hospital, Aarhus, Denmark; 4 Department of Research and Development, Region Kronoberg, Växjö, Sweden; 5 Capio St. Görans Hospital, Stockholm, Sweden; 6 Center for Safety Research, KTH Royal Institute of Technology, Stockholm, Sweden; 7 Cardiology Unit, Karolinska University Hospital, Stockholm, Sweden

**Keywords:** Dysglycaemia, Cardiovascular disease, Inflammation, Complement system proteins, Mannose binding lectin, Biomarker, Prognosis

## Abstract

**Background:**

Mannose binding lectin (MBL) has been suggested to be associated with an impaired cardiovascular prognosis in dysglycaemic conditions, but results are still contrasting.

Our aims are (i) to examine whether MBL levels differ between patients with an acute myocardial infarction (MI) and healthy controls and between subgroups with different glucose tolerance status, and (ii) to investigate the relation between MBL and future cardiovascular events.

**Methods:**

MBL levels were assessed at discharge and after 3 months in 161 AMI patients without any previously known glucose perturbations and in 183 age- and gender-matched controls from the Glucose metabolism in patients with Acute Myocardial Infarction (GAMI) study. Participants were classified as having dysglycaemia, i.e. type 2 diabetes or impaired glucose tolerance, or not by an oral glucose tolerance test. The primary outcome was a composite of cardiovascular events comprising cardiovascular death, AMI, stroke or severe heart failure during 11 years of follow-up. Total and cardiovascular mortality served as secondary outcomes.

**Results:**

At hospital discharge patients had higher MBL levels (median 1246 μg/L) than three months later (median 575 μg/L; p < 0.01), the latter did not significantly differ from those in the controls (801 μg/L; p = 0.47). MBL levels were not affected by dysglycaemia either in patients or controls. Independent of glycaemic state, increasing MBL levels did not predict any of the studied outcomes in patients. In unadjusted analyses increasing MBL levels predicted cardiovascular events (hazard ratio HR: 1.67, 95% confidence interval CI 1.06–2.64) and total mortality (HR 1.53, 95% CI 1.12–2.10) in the control group. However, this did not remain in adjusted analyses.

**Conclusions:**

Patients had higher MBL levels than controls during the hospital phase of AMI, supporting the assumption that elevated MBL reflects acute stress. MBL was not found to be independently associated with cardiovascular prognosis in patients with AMI regardless of glucose state.

**Supplementary Information:**

The online version contains supplementary material available at 10.1186/s12933-022-01562-0.

## Background

Low-grade inflammation is likely involved in the pathogenesis of the cardiovascular complications of dysglycemia, mediated by endothelial dysfunction [1, 2]. Complement activation e.g. via the lectin pathway which is initiated by mannose binding lectin (MBL), has been suggested as a potential key player [3, 4]. MBL acts as an acute phase protein, levels rising up to three-fold during stress [5]. Inter-individual MBL levels range from undetectable to 10,000 μg/L [6] while the genetically-determined intra-individual levels are relatively stable over time [7]. The relationship between MBL levels and macrovascular complications in patients with diabetes is seemingly complex, since some studies reported that low MBL is associated with an impaired cardiovascular prognosis in dysglycaemic conditions, while other reported on the opposite [5]. In a large, prospective Icelandic cohort, low MBL (< 1000 μg/L) was associated with an increased risk of future myocardial infarction independently of other cardiovascular risk factors [7]. By contrast, in the second Diabetes Mellitus Insulin-Glucose Infusion in Acute Myocardial Infarction (DIGAMI 2) trial, comprising patients with type 2 diabetes mellitus and acute myocardial infarction (AMI), low MBL was not independently correlated with cardiovascular outcomes [8]. On the other hand, Hansen et al. [9] showed that high MBL levels in patients with type 2 diabetes mellitus was associated with a significantly increased risk of death after 10 to 15 years. In accordance, a meta-analysis by Zhao et al. [6] collecting data from 12 independent case–control studies, suggested that high expression of MBL may be correlated with a significantly increased risk of vascular complications in diabetes. More recently, a Danish cohort study reported a U-shaped association of MBL with cardiovascular outcomes in patients with type 2 diabetes [10]. Nevertheless, the role of MBL remains to be investigated in controlled settings and possibly related to acute stress.

The aim of this report was (i) to examine whether MBL levels differ between patients with AMI and healthy controls and between subgroups with different glucose tolerance status, and (ii) to investigate the relation between MBL and future cardiovascular events.

## Methods

### Study design

The Glucose metabolism in patients with Acute Myocardial Infarction (GAMI) study aimed at evaluating the prevalence of dysglycaemia in patients with AMI free from known diabetes mellitus [11]. In brief, the study enrolled 181 patients admitted to two Swedish coronary care units for AMI (Karolinska and Västerås hospitals) during 1998–2000 of whom 163 underwent an oral glucose tolerance test (OGTT) before hospital discharge. Exclusion criteria were: known diabetes, serum creatinine ≥ 200 µmol/l, capillary blood glucose ≥ 11.1 mmol/l or age > 80 years [11]. The OGTT disclosed that 65% had newly detected dysglycaemia (defined as diabetes mellitus [48%] or impaired glucose tolerance [IGT; 52%]) according to the World Health Organisation (WHO; Additional Table S1) [12]. Subsequently, gender- and age- matched controls (n = 185) without known diabetes mellitus or cardiovascular disease (CVD) were recruited from the general population between January 2001 and July 2002; OGTT results revealed that 35% of them had dysglycaemia [11].

The present study group comprises GAMI patients with available MBL levels obtained at discharge (n = 161) and three months later (n = 137) and from controls (n = 183) at the baseline visit. Among these, glucose categorization was available in 158 patients at discharge (normal glucose tolerance, NGT = 50, dysglycaemia = 108), in 136 patients three months later (NGT = 44, dysglycaemia = 92) and in all controls (NGT = 119, dysglycaemia = 64).

### Biochemical analyses

*Fasting blood glucose* was analysed in whole capillary blood by means of a HemoCue® portable photometer (HemoCue AB, Ängelholm, Sweden) as soon as possible after hospital admission for patients and at baseline for controls [13]. Venous blood for analysis of glycated hemoglobin A1c (HbA1c) was obtained at admission for patients and at baseline for controls. The analysis was performed by high performance liquid chromatography ion exchange on whole blood applied on filter paper and the values expressed as MonoS standard (Boehringer-Mannheim Scandinavian AB, Bromma, Sweden) [11]. MBL levels were measured using an in-house time-resolved immunofluorometric assay based on the recognition of a ligand combined with detection by a specific monoclonal antibody with the lower detection level of 5 μg/L [14]. The intra-assay and inter-assay coefficients of variation were below 10%. These analyses were performed at the Medical Research Laboratory, Aarhus University Hospital, Denmark.

### Endpoints

The primary endpoint was a composite of cardiovascular events including cardiovascular death, nonfatal AMI, stroke or severe heart failure. Secondary endpoints included all-cause and cardiovascular mortality (caused by AMI, stroke, aortic dissection or sudden death without known reasons).

### Statistical analyses

Continuous variables are presented as median and interquartile range (IQR). Differences between patients and controls were assessed with the Wilcoxon two-sample test. Dichotomous variables are presented as absolute numbers and percentages and compared by chi-square test.

Differences in MBL levels were assessed in patients vs. controls and in NGT vs. dysglycaemia subgroups. Spearman’s test was used to analyse correlations between MBL levels and continuous variables of interest (i.e. age, body mass index—BMI, blood glucose, HbA1c, creatinine, C-reactive protein—CRP and triglycerides) in patients and controls respectively. The Wilcoxon two-sample test was used in order to study differences in MBL levels between groups stratified by dichotomous variables (e.g. female vs. male, and statins vs. no statins).

The relationship between an increase in one standard deviation (SD) of MBL levels and the three endpoints was assessed by Cox regression models, separately in patients and in controls. The SD was calculated separately for each group as following for patients (1332 μg/L) and for controls (962 μg/L). The results are presented as hazards ratio (HR) and 95% confidence intervals (CI).

Kaplan–Meier curves were computed for patients and controls separately, to illustrate time trends for cardiovascular events comparing patients and controls with MBL levels below or above the median value by log-rank test. The statistical significance level was considered at p < 0.05. All statistical calculations were performed in SAS version 9.4.

### Ethics

The GAMI trial complied with the declaration of Helsinki. All patients provided written and oral informed consent. Ethical permission for the original study and the extended follow-up has been obtained from the regional ethics committee of the Karolinska Institutet (Dnr: 98–039, 99–406 and 2011–260-32).

## Results

### Baseline characteristics

Pertinent clinical and biochemical data of the 161 AMI patients and 183 controls are outlined in Table 1. Patients, who had a mean hospital stay of four to five days, were significantly more often smokers and had a more frequent history of other cardiovascular risk factors such as hypertension and hyperlipidaemia. Fasting plasma glucose and HbA1c were significantly higher in patients than controls. After stratifying both patients and controls based on their glycaemic category, newly detected dysglycaemia was approximately twice as common in patients (67%) than in controls (35%).

**Table 1:** Baseline characteristics at admission (patients) and at baseline (controls). Data presented as median and interquartile range (IQR) for continuous variables and as number (%) for dichotomous variables

Variables	Patients n = 161	Controls n = 183	*P*
Clinical characteristics			
Age (years)	63 (57–71)	65 (57–72)	0.26
Female gender	47 (29)	58 (32)	0.64
BMI (kg/m^2^)	26 (24–29)	26 (24–29)	0.69
Current smokers	56 (35)	21 (11)	< 0.01
Family history of diabetes	33 (21)	33 (18)	0.58
Family history of IHD	85 (53)	51 (28)	< 0.01
Previous medical history			
AMI	33 (21)	0 (0)	< 0.01
Stroke	6 (4)	0 (0)	0.01
Congestive heart failure	13 (8)	0 (0)	< 0.01
Hypertension	51 (32)	33 (18)	< 0.01
Hyperlipidaemia	26 (16)	14 (8)	0.02
Pharmacological treatment*			
ACE Inhibitor	16 (10)	10 (5)	0.15
Aspirin	46 (29)	18 (10)	< 0.01
β-Blocker	56 (35)	25 (14)	< 0.01
Ca^2+^ blocker	26 (16)	8 (4)	< 0.01
Thiazide	10 (6)	9 (5)	0.64
Furosemide	15 (9)	10 (5)	0.21
Statins	20 (12)	7 (4)	< 0.01
Biochemical characteristics			
FPG (mmol/L)	6.2 (5.6–7.4)	5.0 (4.6–5.4)	0.02
HbA1c (%)	4.9 (4.6–5.3)	4.6 (4.3–5.0)	< 0.01
eGFR (ml/min/1.73m^2^)	70 (61–82)	N/A	N/A
CRP^2^ (mmol/L)	18 (8–52)	N/A	N/A
Serum triglycerides^ (mmol/L)	1.9 (1.5–2.6)	N/A	N/A
Dysglycaemia^¨^	108 (67)	64 (35)	< 0.01

*P*- values represent significance between groups

### MBL levels

**Fig. 1:**
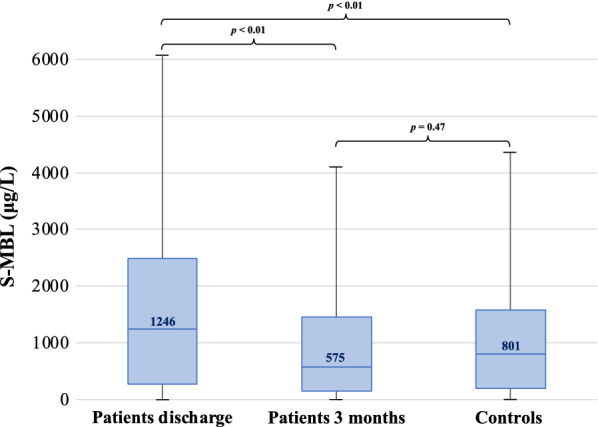
Distribution of serum mannose binding lectin levels (μg/L) in patients at discharge and at three months and in controls at baseline. The bottom of each box represents the 25th percentile, the top the 75th percentile and the line in the middle the median, with the corresponding value; whiskers are minimum and maximum values. *P*-values are displayed for differences between groups. *MBL* mannose binding lectin

As depicted in Fig. 1, median MBL in patients were approximately two times higher at the time of hospital discharge than three months later (*p* < 0.01) and they were significantly higher than in controls (median 1,246 vs. 801 μg/L; *p* < 0.01). There was no significant difference between patients at three months and controls (575 vs. 801 μg/L; *p* = 0.47; Fig. 1).

**Fig. 2:**
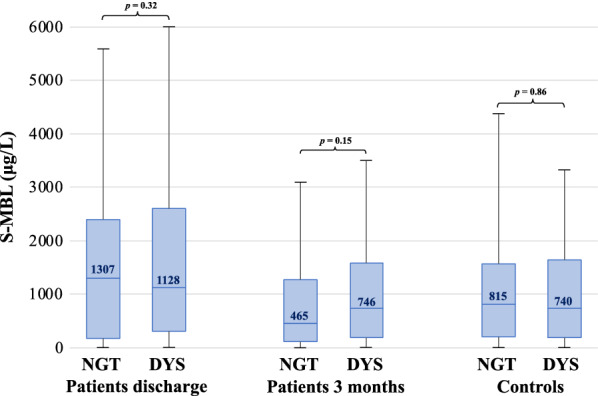
Distribution of serum mannose binding lectin levels (μg/L) in patients at discharge and at 3 months and in controls at baseline, divided by glycaemic status. The bottom of each box represents the 25th percentile, the top the 75th percentile and the line in the middle the median, with the corresponding value; whiskers are minimum and maximum values. *P*-values represent significance between MBL levels in patients and controls with and without dysglycemia respectively. *MBL* mannose binding lectin, *NGT* normal glucose tolerance, *DYS* dysglycaemia

MBL levels in NGT vs dysglycaemia did not differ within groups i.e. within patients at discharge, patients at three months and in controls; Fig. 2).

Table 2 displays MBL levels in patients and controls according to glycaemic status. In patients with dysglycaemia MBL levels were significantly higher at the time of hospital discharge than three months later (*p* < 0.01) and as compared to controls with dysglycaemia (*p* = 0.02). MBL levels did not differ between dysglycaemic patients three months after discharge and dysglycaemic controls (p = 0.98). Patients with NGT had higher MBL levels at discharge than three months later (*p* < 0.01), but there were no statistically significant differences compared with NGT controls (*p* = 0.15 and *p* = 0.16 respectively).

**Table 2:** Mannose-binding lectin levels (μg/L) in patients and controls by glycaemic state, presented as median (interquartile range)

	Patients		Controls
	Discharge (n = 161)	3 months after (n = 137)	Baseline (n = 183)
	NGT (n = 50) DYS (n = 108)	NGT (n = 44) DYS (n = 92)	NGT (n = 119) DYS (n = 64)
Total	1246 (273–2496)	575 (155–1454)	801 (201–1585)
NGT	1307 (188–2405)	465 (115–1275)	815 (207–1571)
DYS	1128 (314–2606)	746 (198–1590)	740 (189–1643)

*NGT* normal glucose tolerance; *DYS* dysglycaemia

In the whole study cohort (patients and controls), there was no significant correlation between MBL and other cardiovascular risk factors, including previous hypertension, myocardial infarction, gender and smoking (data not shown). MBL correlated negatively with HbA1c in patients with NGT (r_s_ =−0.39, *p* < 0.01) and were significantly lower in patients with NGT and statin treatment at discharge than those not on statins (*p* = 0.02). MBL did not correlate with BMI, capillary blood glucose, creatinine, CRP and triglycerides neither in the total patient group nor in subgroups (data not shown). In the control group, there was a weak positive correlation between MBL levels and the age of participants (r_s_ = 0.18, *p* = 0.02). No further significant correlations between MBL and BMI, capillary blood glucose, or HbA1c were found in the total control group.

### MBL and cardiovascular prognosis

The median duration of follow-up was 11.6 years for patients and 10.4 years for controls [15]. As outlined in Table 3, 70 of the patients (43%) and 29 (16%) of the controls suffered the primary composite endpoint. Fifty-one patients (32%) and 27 controls (15%) died. The primary cause of death was cardiovascular in 31 of the patients and 12 of the controls.

**Table 3:** The association of MBL and different outcomes (unadjusted)

	n	Patients n = 161	*P*	N	Controls n = 183	*P*
		HR (95% CI)			HR (95% CI)	
Cardiovascular events^a^	70	1.03 (0.81–1.31)	0.80	29	1.3 (0.93–1.81)	0.13
Cardiovascular mortality	31	0.76 (0.52–1.13)	0.18	12	1.67 (1.06–2.64)	0.03
Total mortality	51	0.89 (0.67–1.18)	0.43	27	1.53 (1.12–2.10)	< 0.01

Increments by one standard deviation (SD) for patients (1332 μg/L) and controls (962 μg/L) from samples at discharge, respectively at baseline

Either cardiovascular mortality, nonfatal AMI, nonfatal stroke or severe heart failure

*n* Data are presented as number of events, *HR* hazard ratio, *CI* 95% confidence interval

^a^Either cardiovascular mortality, nonfatal AMI, nonfatal stroke or severe heart failure

**Fig. 3:**
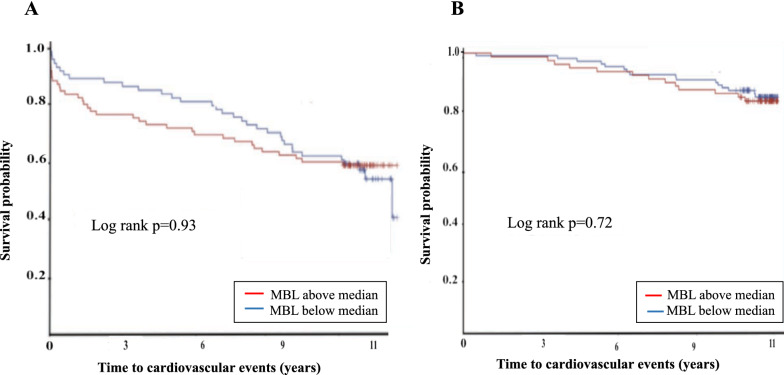
Kaplan–Meier curves in patients (**A**) and controls (**B**) according to serum mannose binding lectin levels below (in blue) or above the median (in red). *MBL* mannose binding lectin

Kaplan–Meier curves for the time to a first cardiovascular event in patients and controls with MBL dichotomized as below or above median are presented in Fig. 3. There was no significant difference in the rate of cardiovascular events neither in patients with MBL below or above the median at discharge (*p* = 0.93; Fig. 3A) nor in controls (*p* = 0.72; Fig. 3B). A similar pattern was seen for cardiovascular and total mortality in patients and controls (data not shown).

The predictive value of MBL for the primary and secondary endpoints is shown in Table 3. In unadjusted Cox regression analyses for the total patient group, an increase by one SD of MBL at the time of hospital discharge did not predict any of the outcomes. This pattern remained when looking separately at patients with and without dysglycaemia, including separate analyses of patients with diabetes and IGT (data not shown). In the control group, an increment of MBL by one SD was associated with cardiovascular mortality (hazard ratio—HR 1.67 [95% CI 1.06–2.64; *p* = 0.03]) and total mortality (HR 1.53 [95% CI 1.12–2.1; *p* < 0.01]) in the unadjusted analyses. However, the association between increasing MBL levels and mortality did not remain after adjustment for age, HbA1c and sex. Similar results concerning the association between increasing MBL levels and mortality were seen in controls with NGT but not in the dysglycaemia subgroup, in which MBL did not predict total or cardiovascular mortality (data not shown). Additional analyses, in each group (patients and controls) separately, using an increase of one unit of MBL did not influence the results.

## Discussion

The major findings are (i) that patients with AMI had the highest MBL levels at discharge while the profile three months later was similar to that of age- and gender-matched controls; (ii) that MBL did not differ between patients and controls with and without dysglycaemia; and (iii) that MBL did not predict future cardiovascular events and total mortality. These findings do not support the assumption that MBL is a pathogenetic factor of relevance for future cardiovascular disease following an AMI in patients with newly diagnosed dysglycaemia.

Since MBL is an acute phase protein it is not surprising that the patients had the highest MBL levels during the immediate post-AMI period, while MBL levels were significantly lower three months later and then comparable to MBL levels among controls. This is compatible with a case–control study [16] where MBL measured within 24 h of an acute coronary syndrome (median 855 μg/L) was significantly higher than in healthy controls and levels obtained three to six months later. Moreover, the trends shown for MBL in the present study are similar to those observed for copeptin in this same GAMI cohort, supporting the hypothesis that it is a stress-related biomarker [17].

An important aspect of the present investigation was to carefully study the relationship between MBL and dysglycaemia by including different levels of dysglycaemia and a control group free from cardiovascular disease to somehow blunt the effect of cardiovascular risk. Interestingly, MBL did not differ comparing patients and controls with and without dysglycaemia. This contrasts with observations from a case–control study by Guan et al. [18] showing that MBL was significantly higher in patients with diabetes mellitus compared with healthy controls. However, in the present study, participants had newly detected dysglycaemia with HbA1c levels within the normal range, while the participants in the study by Guan et al. [18] had a median diabetes duration of 12.5 years and a median HbA1c of 7.0% (HLC-723 G7; TOSHO, Japan, with a normal range of 4–6%). A possible explanation may therefore be that the MBL levels are related to the duration and degree of dysglycaemia. Indeed, hyperglycaemia may lead to glycation of various proteins resulting in autoreactivity of MBL activating the complement system [3]: such mechanism may be relevant in a chronic hyperglycaemic state but may be less apparent when MBL is measured in people with newly detected dysglycaemia as in the present study.

We found no association between increasing MBL levels and cardiovascular prognosis. Previous findings are contrasting, reporting opposite directions for the associations between MBL and cardiovascular disease: either high [9, 16, 19] or low [7] MBL levels have been reported as predictive in diverse populations at different baseline cardiovascular risk, with and/or without diabetes [6]. Differences between these study populations and their characterization might partially explain such contradictions. Moreover, the baseline cardiovascular risk has to be considered: all GAMI patients had a recent AMI, whereas only 10% of the patients studied by Hansen et al. [9] had a previous cardiovascular event and the cohort from the Danish study [10] included a more unselected population with type 2 diabetes. Nevertheless, the hypothesis from the latter study [10], i.e. that MBL levels and cardiovascular risk have a U-shaped relationship, could not be tested in the present study because of the limited sample size.

Indeed, our results are consistent with those of the DIGAMI 2 study, where MBL phenotype and genotype were not independently associated with future cardiovascular events in patients with type 2 diabetes and AMI [8]. This suggests that MBL might be of less importance in patients with already manifest cardiovascular disease while might still bearing prognostic capacity early in the pathophysiological process of cardiovascular disease. This notion gains support from a large prospective study in which MBL was measured in 760 apparently healthy individuals who developed cardiovascular disease during six years of follow-up and in 1,505 matched controls who remained free from it [16]. This is somewhat confirmed by the significant association we found between MBL levels and prognosis in controls, which however disappeared when adjusting for other clinically relevant covariates, possibly for the lack of power.

### Strengths and limitations

The main strength of the present study is that it is based on a carefully characterised cohort of patients with AMI and a well-matched healthy control group. MBL was estimated at two different time points yielding data regarding changes over time in the AMI patients. Moreover, the follow-up time was long, up to 11 years, offering an opportunity to study the impact of MBL on the long-term CVD prognosis. The major limitation is the small sample size causing a potential lack of statistical power due to a relatively low number of events in particular among the controls and in the glycaemic subgroups. Another concern is that it was not possible to obtain MBL blood samples in 24 of the AMI patients three months post-discharge.

## Conclusions

The present findings support the assumption that elevated MBL levels during the hospital phase of an AMI reflects acute stress and do not support that MBL is associated with the cardiovascular prognosis after AMI in patients with newly diagnosed dysglycaemia. Further studies are needed to determine the role of the complement system in the pathophysiology of macrovascular complications in patients with dysglycaemia.

## Supplementary Information

**Additional file 1:** **Table S1. **Definitions of glycaemic categories according to WHO [12]

## Data Availability

The datasets used and/or analysed during the current study are available from the corresponding author on reasonable request.
