# Supplementary material for: Mannose-binding lectin does not explain the dismal prognosis after an acute coronary event in dysglycaemic patients. A report from the GAMI cohort
Source: Cardiovasc Diabetol. 2022 Jul 8;21:129. doi: 10.1186/s12933-022-01562-0 (PMC9270763; doi:10.1186/s12933-022-01562-0)
Supplement: Supplementary file 1 — Additional file 1: Table S1. Definitions of glycaemic categories according to WHO [12] [file 12933_2022_1562_MOESM1_ESM.pdf]

Additional file 1: Table S1. Definitions of glycaemic categories according to WHO [12]

	Venous plasma glucose (mmol/L)	
	**Fasting venous**	**2-hour post-load**
**Normoglycaemic**	<6.1	<7.8
**Impaired fasting glucose (IFG)**	6.1 – 6.9	< 7.8
**Impaired glucose tolerance (IGT)**	<7.0	7.8–11.0
**Diabetes**	≥7.0	>11.0
